# Superb microvascular imaging evaluating joint lesion scores in rheumatoid arthritis compared with power Doppler imaging: A meta-analysis: Retraction

**DOI:** 10.1097/MD.0000000000042732

**Published:** 2025-06-06

The article, “Superb microvascular imaging evaluating joint lesion scores in rheumatoid arthritis compared with power Doppler imaging: A meta-analysis”,^[1]^ which appears in Volume 99, Issue 37 of *Medicine*, is being retracted over concerns with the methodology. After publication, concerns were raised over references to the “Begger test,” which does not exist in the literature and is indicative of possible fraud. Upon further investigation, authorship irregularities were also discovered in our submission system. As a result, the Publisher no longer has confidence in the integrity of the content or the authorship.
